# Yellow and purple nutsedge and coffee senna as hosts of common plant nematodes in Florida

**DOI:** 10.21307/jofnem-2020-094

**Published:** 2020-11-24

**Authors:** Maria de Lourdes Mendes, Donald W. Dickson, William T. Crow

**Affiliations:** Entomology and Nematology Department, University of Florida, PO Box 110620, Gainesville, FL, 32611

**Keywords:** *Belonolaimus longicaudatus*, Coffee senna, *Cyperus*, Host-status, *Meloidogyne*, Purple nutsedge, Root-knot nematode, *Senna occidentalis*, Sting nematode, Weed, Yellow nutsedge

## Abstract

Yellow (*Cyperus esculentus*) and purple (*C. rotundus*) nutsedges, and coffee senna (*Senna occidentalis*) are common weeds in the southern USA and each have been reported as alternative hosts for plant-parasitic nematodes. Our objective was to determine the host suitability of these weeds to plant-parasitic nematodes common in Florida agriculture and turfgrass systems. The root-knot nematode (RKN) species tested included *Meloidogyne arenaria*, *M. enterolobii*, *M. floridensis*, *M. graminis*, *M. hapla*, *M. incognita*, and *M. javanica*. The host status of sting nematode, *Belonolaimus longicaudatus*, was also evaluated, but only on the nutsedge species. All RKN species evaluated reproduced on both nutsedge species and had a reproductive factor greater than one, except for *M. graminis* on yellow nutsedge. However, only *M. hapla*, *M. javanica*, and *M. graminis* induced visual galls on yellow nutsedge and only *M. graminis* caused galling on purple nutsedge. *Meloidogyne arenaria* and *M. graminis* reproduced at a greater rate on purple nutsedge than on yellow nutsedge. Both nutsedge species were good hosts to *B. longicaudatus*. Coffee senna was a host to *M. enterolobii*, a poor host to *M. incognita*, and nonhost to the other RKN species evaluated.

Yellow and purple nutsedges (*Cyperus esculentus* and *C. rotundus*, respectively) are among the worst weeds affecting agriculture production worldwide (Peerzada, 2017). In Florida, nutsedges are highly damaging and ubiquitous weeds in almost every agricultural and horticultural production system. Coffee senna, or coffee weed (*Senna occidentalis* syn. *Cassia occidentalis*) is a leguminous annual weed found in tropical and subtropical regions worldwide (CABI, 2016). In the United States, coffee senna is a common weed in agronomic crops and is particularly important because its seeds contain animal toxins (Furlan et al., 2012).

Weeds often serve as alternative hosts to plant-parasitic nematodes, thereby increasing their incidence and severity. In addition, they reduce the efficacy of nematode management tactics. For example, nutsedges and plant-parasitic nematodes are two of the most common soilborne pest problems in Florida vegetable production and often occur concomitantly (Rich et al., 2003). Yellow and purple nutsedges are both hosts to *Meloidogyne incognita* (Schroeder et al., 1993) and can harbor life stages of the nematode within their tubers. Consequently, fumigant nematicides fail to kill many of the individuals housed within tubers (Thomas et al., 2004). Yellow nutsedge is reported to be a host to *M. graminicola* and *Hoplolaimus columbus* (Bird and Hogger, 1973; Minton et al., 1987; Schroeder et al., 1993), whereas purple nutsedge is reported to be a host to *M. graminicola*, *H. columbus*, *Belonolaimus longicaudatus*, *Dolichodorus heterocephalus*, *Nanidorus minor*, and *Ditylenchus destructor* (Rhoades, 1964; Bird and Hogger, 1973; Minton et al., 1987; De Waele et al., 1990; Schroeder et al., 1993). Furthermore, nutsedge tuber counts were highly correlated with soil infestation densities of *M. incognita* (Thomas et al., 1995; Ou et al., 2008).

In Florida’s high-value vegetable and strawberry production systems nutsedges are a primary weed problem, and root-knot nematodes (RKN) of various species and sting nematode (*B. longicaudatus*) are the nematodes of greatest concern (Kokalis-Burelle, 2003; Khanal and Desaeger, 2020). *Meloidogyne incognita, M. javanica, M. arenaria, M. enterolobii*, and *M. floridensis* are the most common RKN reported in Florida vegetable fields (Brito et al., 2008), whereas *M. hapla* is second only to sting nematode in importance on strawberry (Desaeger, 2019). Bermudagrass sod farms, golf courses, athletic fields, lawns, and pastures are often infested with nutsedges that may serve as alternative hosts to sting nematode and the grass RKN *M. graminis*, the two most damaging plant-parasitic nematodes on this plant.

Coffee senna is a common weed in cotton and peanut production in northern Florida, as are yellow and purple nutsedges. *Meloidogyne incognita* and *Rotylenchulus reniformis* on cotton, and *M. arenaria* on peanut, are the most important nematodes on these crops in the region. Coffee senna was reported as a host to *R. reniformis* (Lawrence et al., 2008), whereas it was a nonhost to *M. arenaria*, *M*. *enterolobii*, *M. floridensis*, *M. incognita*, and *M. javanica* (Kaur et al., 2007).

In order to improve the effectiveness of nematode management strategies it is very important to know the host status of common weeds to the nematode species present in agricultural fields. The objective of this study was to determine the host suitability of yellow and purple nutsedges and coffee senna to some of the common plant-parasitic nematodes important to Florida agriculture and turfgrass production systems. To meet this objective, three experiments were conducted under greenhouse conditions and each was repeated.

## Material and methods

### Host status of common weeds to *Meloidogyne* spp.

We evaluated the host status of yellow and purple nutsedge and coffee senna to *Meloidogyne arenaria*, *M. enterolobii, M. floridensis, M. hapla, M. incognita,* and *M. javanica*. ‘AgriSet 334’ tomato (*Solanum lycopersicum*) was included as a susceptible control. This experiment was originally conducted in summer of 2014 (trial 1) and repeated in summer of 2015 (trial 2). There were five replications of the 18 RKN species × host plant combinations placed on a greenhouse bench in a completely randomized design. Six tubers of either of yellow (*C. esculentus*) or purple nutsedge (*C. rotundus*) from greenhouse cultures were sown in clay pots (16.0-cm-top outside diam.) containing 1,400 cm^3^ of steam pasteurized soil (92% sand, 3% silt, 5% clay, and 1% organic matter). Coffee senna and tomato were pre-germinated in a vermiculite medium and transplanted to the clay pots 3 weeks later. Nematode inocula were prepared by extracting eggs from infected tomato root systems from greenhouse cultures using the technique of Hussey and Barker (1973), as modified by Bonetti and Ferraz (1981). The test plants, 12-week old nutsedge and 6-week old tomato and coffee senna seedlings, were inoculated with 5,000 eggs and second-stage juveniles (J2) of the respective RKN species. Inoculated plants were maintained under greenhouse conditions, greenhouse temperatures for this experiment, and the subsequent experiments, ranged from 23 to 31°C, with an average temperature of 27°C. At 85 days after inoculation the plant roots were removed from pots, thoroughly washed to remove soil and debris, fresh root weights recorded, and any visible galls rated by using the 0 to 5 scale (Taylor and Sasser, 1978). Eggs were extracted as described above to determine the reproductive factor (Rf) for each nematode species.

### Host status of nutsedges to *Meloidogyne graminis*

This experiment assessed the host suitability of yellow and purple nutsedges to the grass RKN, *M. graminis*. ‘Tifway’ bermudagrass was used as a known susceptible control (W. T. Crow, pers. comm.). This experiment was originally conducted in spring-summer of 2016 (trial 1) and repeated in spring-summer of 2018 (trial 2). There were five replications of three different host plant treatments placed on a greenhouse bench in a completely randomized design. Clay pots (16.0-cm-top outside diam.) containing 1,400 cm^3^ of sterilized sand were planted with six tubers of either yellow or purple nutsedge, or sprigs of bermudagrass. Twelve-week-old nutsedge and 8-week-old bermudagrass were inoculated with 4,000 J2 of *M. graminis* per pot. To collect inoculum, infected bermudagrass roots from greenhouse cultures were incubated in a mist chamber for a period of 72 hr (Crow et al., 2020) to collect J2 exiting roots. After inoculation plants were maintained under glasshouse conditions as described above. In total, 150 days after inoculation the plants were uprooted and nematode population density was assessed along with fresh root and tuber weights. Vermiform J2 and males were extracted separately from the entire pot contents of roots and tubers by using the mist extraction method for a period of 72 hr (Crow et al., 2020). Before the incubation in the mist chamber, the tubers were superficially disinfected with 0.5% sodium hypochlorite for 20 min to remove live nematodes from tuber surfaces. Because *M. graminis* is a bisexual species with abundant males, J2 and male life stages were counted separately; however, only J2 were used to calculate the Rf.

### Host status of nutsedges to *Belonolaimus longicaudatus*

This experiment was originally conducted in spring of 2017 (trial 1) and repeated in winter of 2018 to 2019 (trial 2). ‘FX 313’ St. Augustinegrass (*Stenotaphrum secundatum*) was used as a known host to *B. longicaudatus* (Busey et al., 1991). Clay pots (16.0-cm-top outside diam.) containing 1,400 cm^3^ of sterilized sand were planted with six tubers of either yellow or purple nutsedge, or sprigs of St. Augustinegrass. Twelve-week-old nutsedge and 6-week-old St. Augustinegrass were inoculated with 120 mixed-life stages (J2, J3, J4, male, and female) of *B. longicaudatus* per pot. One hundred eighty days after inoculation the nematode population density was recorded. In trial 1 sting nematodes were extracted from 200 cm^3^ of soil using a sieving and incubation method (McSorley and Frederick, 1991), and in trial 2 they were extracted using the centrifugal-flotation method (Jenkins, 1964). The nematodes recovered from 200 cm^3^ of soil were multiplied by seven to estimate the total number of nematodes per pot (1,400 cm^3^ of soil) for calculating the Rf.

### Statistical analysis

For all experiments data were subjected to analysis of variance and treatment means were separated according to Duncan’s multiple range test (*P* ≤ 0.05) using SAS software (SAS Institute, Cary, NC). When no significant trial or trial × treatment effects occurred the data from the two trials were combined for analysis, otherwise the results of the two trials were analyzed separately.

## Results

### Host status of common weeds to *Meloidogyne* spp.

**Figure 1:**
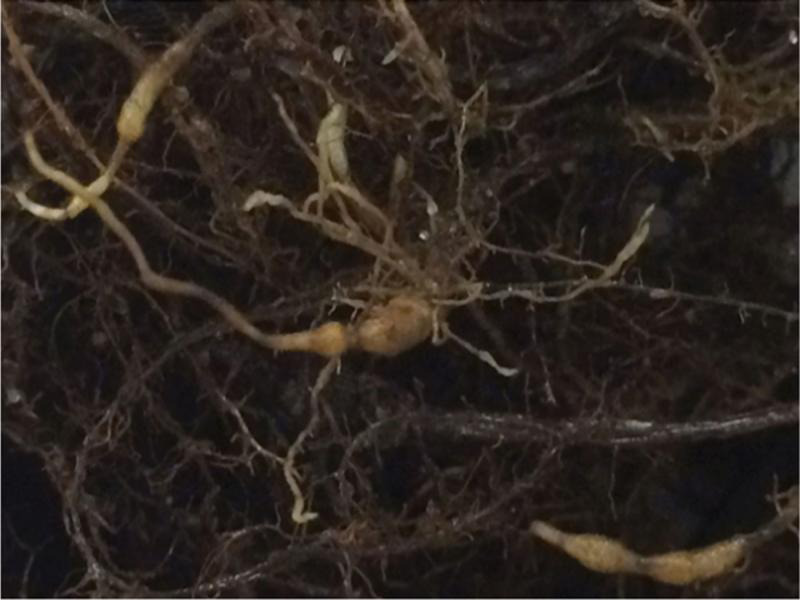
Galling of yellow nutsedge (*Cyperus esculentus*) roots induced by *Meloidogyne javanica*.

Because of significant trial and trial × treatment effects, the data from the two trials were subjected to separate statistical analysis. In the first trial all of the yellow nutsedge plants and three of the purple nutsedge plants inoculated with *M. hapla* died before termination. As plants died their roots were inspected for galling, these measurements were noted but not included in the statistical analysis. Yellow and purple nutsedges were good hosts for all six RKN species (Table 1). The Rf of *M. floridensis* in trial 1 was the highest (*P* ≤ 0.05) among the nematode species on yellow and purple nutsedges with the exception of being similar to *M. arenaria* on purple nutsedge. In trial 2, *M. floridensis* had similar (*P* ≤ 0.05) Rf values on yellow nutsedge to that of *M. hapla* and *M. javanica*, and again on purple nutsedge both *M*. *floridensis* and *M. arenaria* had higher Rf values among the *Meloidogyne* spp. tested. In both trials, the Rf of *M. arenaria* was higher (*P* ≤ 0.05) on purple nutsedge than on yellow nutsedge, whereas it was reversed for *M. enterolobii*. *Meloidogyne incognita* and *M. javanica* had a similar Rf value on both nutsedges in trial 1; however, in trial 2 both reproduced more readily (*P* ≤ 0.05) on yellow nutsedge. Only *M. hapla* (trial 2 only) and *M. javanica* (Fig. 1) induced visible galls on yellow nutsedge and no galls were visible on purple nutsedge (Table 1).

**Table 1.** Reproductive factor (Rf) and gall ratings of *Meloidogyne arenaria*, *M. enterolobii*, *M. floridensis*, *M. hapla*, *M. incognita*, and *M. javanica* on yellow and purple nutsedges, coffee senna, and a known susceptible host ‘AgriSet 334’ tomato in two trials of a greenhouse experiment 84 days after plants were inoculated with 5,000 eggs and second–stage juveniles.

Nematode sp.	Y. nutsedge	P. nutsedge	C. senna	Tomato	Y. nutsedge	P. nutsedge	C. senna	Tomato
	Rf				Gall index (0-5)			
		Trial 1						
*M. arenaria*	2.82 c BC^1^	4.76 b A	0.00 d B	7.75 a A	0.00 b B	0.00 b A	0.00 b C	5.00 a A
*M. enterolobii*	3.93 b B	1.47 c B	1.89 c A	6.88 a A	0.00 b B	0.00 b A	4.60 a A	5.00 a A
*M. floridensis*	5.27 ab A	3.69 b A	0.00 c B	6.66 a A	0.00 b B	0.00 b A	0.00 b C	5.00 a A
*M. hapla*	–	1.57 b B	0.00 c B	2.90 a B	–	0.00 b A	0.00 b C	5.00 a A
*M. incognita*	2.40 b C	1.61 b B	0.08 c B	6.73 a A	0.00 c B	0.00 c A	4.00 b B	5.00 a A
*M. javanica*	2.11 b C	1.72 b B	0.00 c B	8.24 a A	3.00 b A	0.00 c A	0.00 c C	5.00 a A
		Trial 2						
*M. arenaria*	3.10 c C	6.75 b A	0.00 d B	49.30 a A	0.00 b B	0.00 b A	0.00 b C	5.00 a A
*M. enterolobii*	5.95 b B	1.83 c B	1.77 c A	24.20 a C	0.00 c B	0.00 c A	4.40 b A	5.00 a A
*M. floridensis*	8.93 b A	6.06 b A	0.00 c B	35.99 a B	0.00 b B	0.00 b A	0.00 b C	5.00 a A
*M. hapla*	6.83 a AB	1.59 b B	0.00 b B	7.80 a E	0.80 b B	0.00 c A	0.00 c C	5.00 a A
*M. incognita*	5.46 b B	1.44 c B	0.07 c B	15.45 a D	0.00 c B	0.00 c A	3.00 b B	5.00 a A
*M. javanica*	8.97 b A	2.28 c B	0.00 c B	32.05 a B	1.80 b A	0.00 c A	0.00 c C	5.00 a A

**Notes:** ^1^Data are means of five replications except in the *M. hapla* treatment of trial 1 where all plants died in the yellow nutsedge pots and three out of five plants died in the purple nutsedge pots. Treatment means within the same row of each parameter with common lower case letters are not different according to Duncan’s multiple-range test (*P* ≤ 0.05). Treatment means within the same column in the same trial with common upper case letters are not different according to Duncan’s multiple-range test (*P* ≤ 0.05).

**Figure 2:**
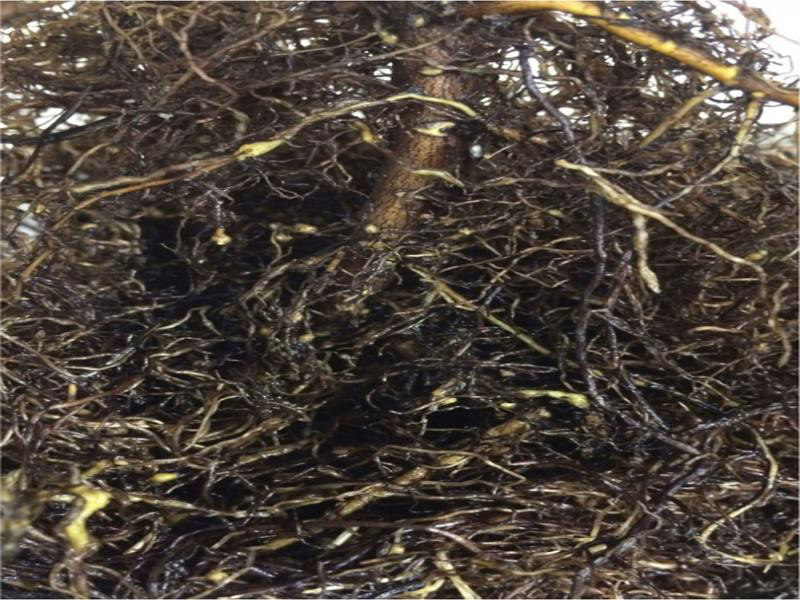
Galling of coffee senna (*Senna occidentalis*) roots induced by *Meloidogyne enterolobii*.

Coffee senna was a good host to *M. enterolobii*, a poor host to *M. incognita*, and a nonhost to the other RKN species. *Meloidogyne enterolobii* (Fig. 2) and *M. incognita* were the only RKN species to induce galls on coffee senna.

### Host status of nutsedges to *Meloidogyne graminis*

**Figure 3:**
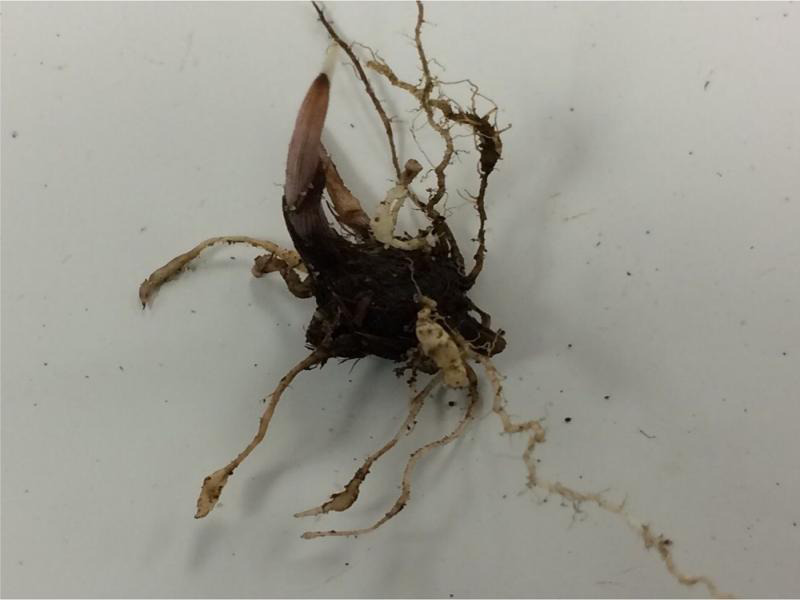
Galling of purple nutsedge (*Cyperus. rotundus*) roots induced by *Meloidogyne graminis*.

The data from the two trials were not heterogeneous and were combined for analysis. Purple nutsedge was a good host, and yellow nutsedge a poor host to *M. graminis* (Table 2). The number of J2 and males recovered from purple nutsedge roots was lower (*P* ≤ 0.05) than from bermudagrass, however, the Rf was not statistically different. *Meloidogyne graminis* was recovered from tubers of yellow and purple nutsedges, although recovery of J2 and males was higher (*P* ≤ 0.05) from purple nutsedge than from yellow nutsedge. Galling caused by *M. graminis* was observed on both nutsedge species (Fig. 3).

**Table 2.** *Meloidogyne graminis* second-stage juveniles (J2) and males per gram of root, per gram of tuber, and reproductive factor (Rf) on yellow nutsedge and purple nutsedge, and the susceptible host ‘Tifway’ bermudagrass in a greenhouse experiment 150 days after plants were inoculated with 4,000 J2.

Host	J2/g root	Males/g root	J2/g tuber	Males/g tuber	Rf^a^
Yellow nutsedge	26b^b^	<1b	1b	<1b	0.35b
Purple nutsedge	240b	<1b	5a	<1a	2.48a
Bermudagrass	509a	24a	–	–	3.64a

**Notes:** ^a^Rf is based on number of J2/pot recovered after incubation of the total root system in a mist chamber for 72 hr; ^b^Data from two trials are combined for analysis. Treatment means within the same column followed by common letters are not different according to Duncan’s multiple-range test (*P* ≤ 0.05).

### Host status of nutsedges to *Belonolaimus longicaudatus*

Because of large variability between the two trials resulting from using different extraction methods, the data from each trial were subjected to statistical analysis separately (Table 3). Both yellow and purple nutsedges had similar (*P* ≥ 0.05) Rf values in both trials to that of the susceptible host ‘FX-313’ St. Augustinegrass. Although not evident in trial 1, Rf values from trial 2 suggested that both nutsedge species were good hosts for *B. longicaudatus*.

**Table 3.** Number of *Belonolaimus longicaudatus* extracted from soil collected from pots containing yellow nutsedge and purple nutsedge, and the known susceptible host ‘FX-313’ St. Augustinegrass, and the reproductive factor (Rf) in two trials of a greenhouse experiment 180 days after being inoculated with 120 mixed-life stages of *B. longicaudatus* per pot.

	Trial 1^a^		Trial 2	
Host	Nematodes/pot	Rf	Nematodes/pot	Rf
Yellow nutsedge	21a^b^	0.16a	228a	1.90a
Purple nutsedge	23a	0.19a	375a	3.12a
St. Augustinegrass	25a	0.21a	389a	3.24a

**Notes:** ^a^Trial 1, nematodes were extracted from soil using a sieving and incubation method. Trial 2, nematodes were extracted using a centrifugal-flotation method. ^b^Data are means of five replications. Treatment means within the same column followed by common letters are not different according to Duncan’s multiple-range test (*P* ≤ 0.05).

## Discussion

Previous experiments have evaluated the host status of either yellow or purple nutsedges against certain RKN species and sting nematode; however, this is the first time that both nutsedge species have been evaluated as hosts for all the nematodes mentioned herein. Because both species of nutsedge were hosts to all the RKN species common in Florida and sting nematode, nutsedge management obviously plays an important role in determining nematode population densities for most Florida crops. Results from this study suggest the importance of implementing effective management practices for both nutsedges in any crop rotation scheme.

In trial 1 of the experiment evaluating the host status of common weeds to *Meloidogyne* spp. experiment, a bioassay was performed to confirm transmission of RKN via nutsedge tubers. Tubers of both yellow and purple nutsedge, previously inoculated with each nematode species, were superficially disinfected by soaking them in 0.5% sodium hypochlorite for 20 min and then rinsed with running water to eliminate the sodium hypochlorite residue. Six tubers were randomly selected were sown in clay pots containing pasteurized field soil, and a tomato seedling was planted into each pot, with six pots from every RKN species. Subsequent tomato root galling was observed for every RKN species (data not shown), indicating that each species was readily transmitted via tubers.

Knowledge that tubers of both nutsedges harbor life stages of RKN species is especially important because once the nematodes are inside tubers they are shielded from soil fumigants as has been previously reported (Thomas et al., 2004). The phase out of methyl bromide has resulted in growers reliance on fumigants (1,3-dichloropropene, metam-sodium, chloropicrin, or combinations) less effective than methyl bromide for nutsedge management (Unruh et al., 2002; Rich et al., 2003; Boyd and MacRae, 2018). Consequently, nutsedge tubers become a primary source of RKN reinfestation in fumigated fields for high-value crops. This is a possible explanation for why RKN damage become so severe on a double crop following a primary crop during a growing season in Florida. Furthermore, 1,3-dichloropropene is the primary fumigant nematicide used for row crops in Florida and is used as a post-plant nematicide on turfgrasses in Florida. Thus, nutsedge management becomes a critical component for any RKN management plan in Florida row crops. In our trials, each RKN species was readily transmitted via tubers to tomato confirming the importance of tubers housing and protecting life stages of RKN from the soil fumigant.

In recent years, root-knot nematodes have become an increasing management problem on golf course bermudagrass (Crow et al., 2020). It is likely that since metam sodium has become the predominant soil fumigant used in sod fields following the loss of methyl bromide, lack of nutsedge management with metam sodium and corresponding *M. graminis* survival within tubers might be a contributing factor to this problem.

While most root-knot nematode species evaluated reproduced well on yellow and purple nutsedge, only *M. graminis*, *M. hapla*, and *M. javanica* induced visible galls on the former and only *M. graminis* induced visible galls on purple nutsedge. Similarly, *M. incognita* caused galling on coffee senna, but did not reproduce well on this plant. It is common in literature to see root galling used as a means to measure the degree of plant resistance or even as a means to determine the extent of nematode reproduction. Results from this study suggest that relying solely on root gall index as a measurement of RKN infection may lead to false conclusions.

Coffee senna is not a good host to the RKN species (*M. incognita* and *M. arenaria*) most common on cotton and peanut in the southeast region, and is not likely to be a major factor in RKN management in these crops. However, coffee senna was a host to *M. enterolobii*, and this could become an issue as this invasive RKN species becomes more widespread. Schwarz et al. (2020) found *M. enterolobii* in sweet potato, soybean, and tobacco fields, indicating this nematode is spreading to agronomic crops. Our results differed from Kaur et al. (2007) who reported coffee senna was a nonhost to a different isolate of *M. enterolobii*, suggesting that host-races of *M. enterolobii* may exist.

Low recovery of *B. longicaudatus* was obtained in the trial when a sieving-incubation extraction method was used. This extraction method was chosen based on the report that it provided good recovery of sting nematodes (McSorley and Frederick, 1991). In the interval between the two trials we conducted our own method comparisons and found low recovery of *B. longicaudatus* using this method compared to centrifugal-flotation (unpublished data), therefore, we changed the extraction method for trial 2. While the resulting numbers varied greatly between trials, the Rf on both nutsedge species was similar to that on the known susceptible host in both trials. Therefore, we conclude that both yellow and purple nutsedges are good hosts despite having an Rf  > 1 only in the second trial.

Purple nutsedge has been reported as a good host to sting nematode (Rhoades, 1964), but this is the first time that the host status of yellow nutsedge to sting nematode has been evaluated and shown to be a good host. Being an ectoparasite, sting nematode is not likely to be protected from soil fumigants inside of tubers similar to that which occurs with RKN; however, yellow and purple nutsedge management during the crop off-season may be very important for management of this devastating nematode pest. High-value crops, namely strawberry, tomato, and melons are grown in the winter in most of Florida and fields are often left fallow during the summer. If nutsedges are allowed to proliferate during the summer months they likely increase the population density of sting nematode and increase the need for, and decrease the efficacy of, nematicide treatments.
